# GH-Producing Pituitary Adenoma and Concomitant Rathke's Cleft Cyst: A Case Report and Short Review

**DOI:** 10.1155/2015/948025

**Published:** 2015-03-25

**Authors:** Ryota Tamura, Satoshi Takahashi, Katsura Emoto, Hideaki Nagashima, Masahiro Toda, Kazunari Yoshida

**Affiliations:** ^1^Department of Neurosurgery, Keio University Hospital, 35 Shinanomachi, Shinjuku-ku, Tokyo 160-8582, Japan; ^2^Division of Diagnostic Pathology, Keio University Hospital, 35 Shinanomachi, Shinjuku-ku, Tokyo 160-8582, Japan

## Abstract

Concomitant pituitary adenoma (PA) and Rathke's cleft cyst (RCC) are rare. In some cases, such PA is known to produce pituitary hormones. A 53-year-old man was admitted to our hospital with a diagnosis of lacunar infarction in the left basal ganglia. Magnetic resonance imaging (MRI) incidentally showed a suprasellar mass with radiographic features of RCC. When he consulted with a neurosurgical outpatient clinic, acromegaly was suspected based on his appearance. A diagnosis of growth hormone- (GH-) producing PA was confirmed from hormonal examinations and additional MRI. Retrospectively, initial MR images also showed intrasellar mass that is compatible with the diagnosis of PA other than suprasellar RCC. The patient underwent endonasal-endoscopic removal of the PA. Since we judged that the RCC of the patient was asymptomatic, only the PA was completely removed. The postoperative course of the patient was uneventful and GH levels gradually normalized. Only 40 cases of PA with concomitant RCC have been reported to date, including 13 cases of GH-producing PA. In those 13 cases, RCC tended to be located in the sella turcica, and suprasellar RCC like this case appears rare. In a few cases, concomitant RCCs were fenestrated, but GH levels normalized postoperatively as in the cases without RCC fenestration. If radiographic imaging shows typical RCC, and PA is not obvious at first glance, the possibility of concomitant PA still needs to be considered. In terms of treatment, removal of the RCC is not needed to achieve hormone normalization.

## 1. Introduction

The relationship between pituitary adenoma (PA) and Rathke's cleft cyst (RCC) is controversial. The origin of RCC is generally considered to be derived from remnants of Rathke's pouch. PA is also formed by proliferation of the anterior wall of Rathke's pouch. They have a possibility to be derived from a common ancestry [1]. It has also been thought that they were derived from “transitional” cells between the lining of Rathke's cleft and the glandular cells of the anterior pituitary. This theory is based on the fact that PA occasionally contains both elements of fetal Rathke's pouch and differentiated adenohypophyseal cells [2]. RCC is reported to be found incidentally in 11–33% of postmortem examinations [1], but concomitant PA and RCC are extremely rare. In some cases, such PA is known to produce various pituitary hormones. We report herein a rare case of growth hormone- (GH-) producing PA with concomitant RCC.

## 2. Clinical Presentation

### 2.1. Onset and Course

**Figure 1:**
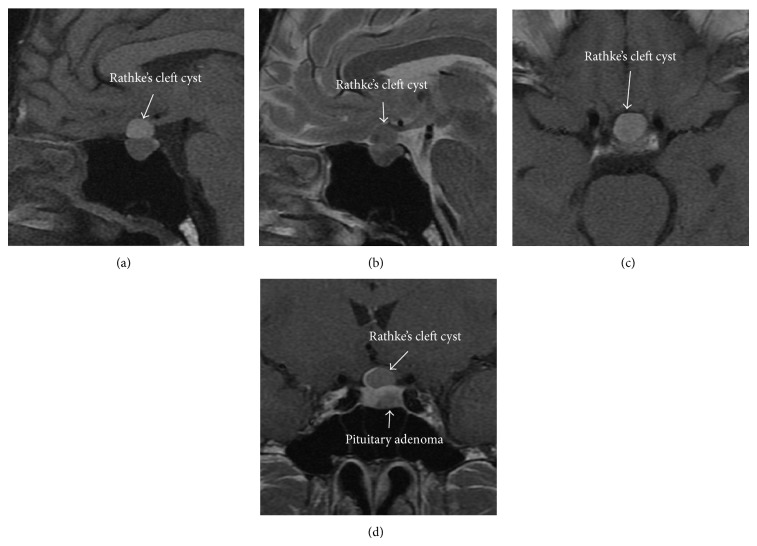
(a) Sagittal T1-weighted MRI of the head shows a suprasellar, high-intensity mass suspected to represent RCC. PA located below the RCC shows isointensity. (b) Sagittal T2-weighted MRI of the head shows isointense RCC and isointense PA. (c) Contrast-enhanced axial MRI shows nonenhancing RCC. In contrast, the normal pituitary gland shows strong enhancement. (d) Contrast-enhanced coronal MRI shows slight compression of the optic chiasma by RCC. And it shows an intrasellar PA of 9 mm in diameter located on the left of normal gland and suprasellar RCC of 12 mm in diameter that compressed stalk to the right side.

**Figure 2:**
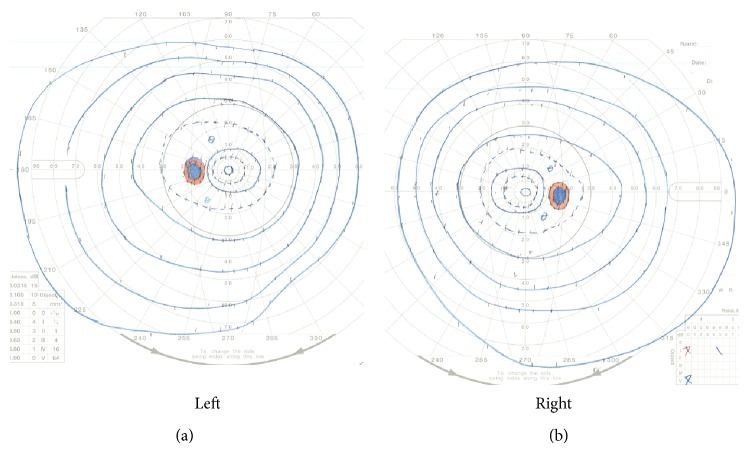
No visual disturbance was apparent.

A 53-year-old man had high height from the cradle and also realized his protruding chin. He presented to our hospital with slight paralysis of the right upper extremity. His height was 181 cm that was within the limits of 2 standard deviations. Head MRI showed left lacunar infarction of the basal ganglia, as well as a suprasellar mass. The suprasellar mass was hyperintense on T1-weighted MR image (Figure 1(a)) and also isointense on T2-weighted MR image (Figure 1(b)). These findings for the suprasellar mass were compatible with RCC, and he was referred to the neurosurgical outpatient clinic for management. At the neurosurgical outpatient clinic, neurological examination revealed only slight right hemiparesis due to lacunar infarction. No visual disturbance was apparent (Figures 2(a) and 2(b)). Physical examination revealed typical features of acromegaly, such as soft tissue swelling visibly resulting in enlargement of the feet, pronounced brow protrusion, and enlargement of the tongue and teeth spacing. At this time, MRI was reviewed by a neurosurgeon, and an intrasellar mass other than the suprasellar lesion was identified. The patient underwent hormonal laboratory testing as well as MRI with intravenous infusion of gadolinium (Gd). Hormonal laboratory testing showed GH of 8.4 ng/mL, somatomedin C of 607 ng/mL (85~240 ng/mL), FSH of 7.9 mIU/mL, LH of 2.2 mIU/mL, testosterone of 4.43 ng/mL, TSH of 2.2 *μ*IU/mL, FT3 of 3.2 pg/mL, FT4 of 1.8 ng/mL, serum cortisol of 14.5 *μ*g/dL, and serum prolactin of 17.5 ng/mL.

Pituitary insufficiency is common at presentation of RCC; however pituitary function status was normal in this case [3].

A 75 g oral glucose tolerance test failed to suppress GH to <1 ng/mL. Nadir GH during this test was 8.3 ng·mL. In conjunction with the findings from Gd-enhanced MRI, a diagnosis of GH-producing PA was made (Figures 1(c) and 1(d)). At this time, we diagnosed GH-producing PA with concomitant RCC. Endonasal-endoscopic approach for removal of the PA was proposed, with the aim of normalizing GH levels. Before surgery, we also planned to fenestrate the RCC only if this could be achieved without difficulty.

### 2.2. Operation

**Figure 3:**
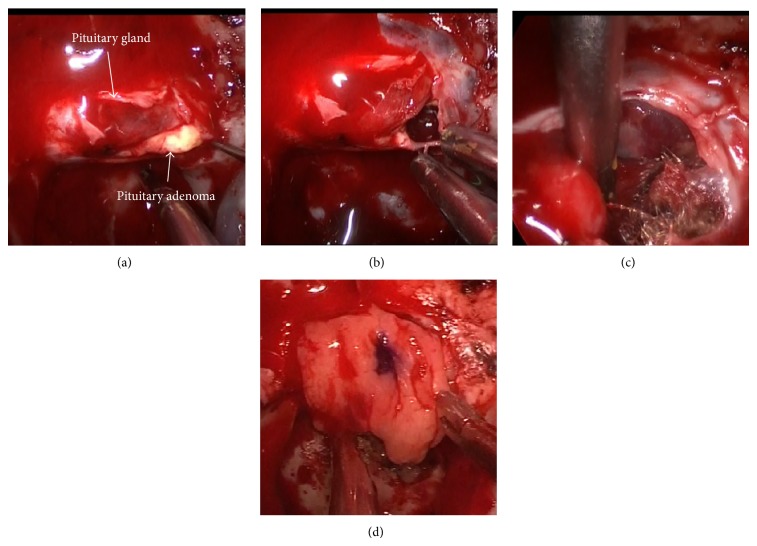
(a) Neuroendoscope shows the clear margin between normal gland and PA. PA looks soft and yellowish. (b) Neuroendoscopic view after removal of the PA, which was easy to remove. (c) Neuroendoscope shows the wall of the RCC. Resecting the suprasellar RCC while retracting normal gland was difficult. (d) The floor of the sella turcica was reconstructed using fat tissue. We did not aspirate or resect the cyst wall.

**Figure 4:**
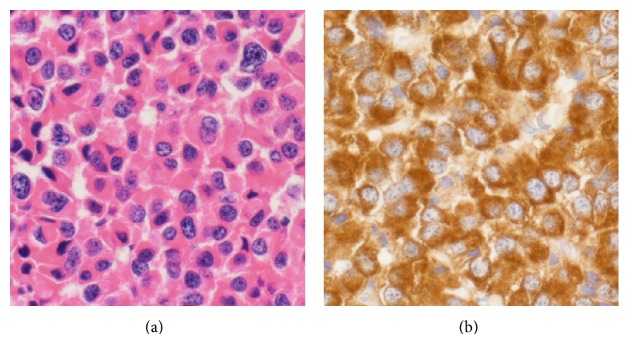
(a) Photomicrograph shows the tumor is composed of monotonous eosinophilic cells. (H&E stain, original magnification ×400.) (b) Positive staining is observed with GH immunohistochemistry. (GH, original magnification ×400.)

Removal of the PA using an endonasal-endoscopic approach was performed. Intraoperatively, the margin between the normal pituitary gland and adenoma was clear (Figure 3(a)). We identified the yellowish adenoma and completed gross total removal using suction (Figure 3(b)). We then retracted the pituitary gland and identified the wall of the RCC (Figure 3(c)). We did not aspirate or resect the cyst wall for fear of cerebrospinal fluid (CSF) leakage. The floor of the sella turcica was reconstructed using fat tissue (Figure 3(d)). Histologically, the tumor was composed of monotonous eosinophilic cells (Figure 4(a)). Positive staining was observed with GH immunohistochemistry (Figure 4(b)); thus the tumor was diagnosed as GH-producing PA. Postoperative computed tomography revealed gross total removal of the adenoma.

### 2.3. Postoperative Course

Postoperative course of the patient was uneventful other than diabetes insipidus, which was well controlled by medication. GH and somatomedin C levels gradually improved until 7 days postoperatively. We decided to observe the RCC conservatively without additional surgery. The patient was discharged from our hospital on foot with no neurological sequelae.

Diabetes insipidus was treated by administered medication for 6 months. Pituitary function and visual field were not disturbed for 6 months. Hormonal laboratory testing 12 weeks after the operation showed GH of 0.3 ng/mL and somatomedin C levels of 131 ng/mL. At the 6-month follow-up time point, he has made satisfactory progress without recurrence.

## 3. Discussion

A great variety of lesions such as sarcoidosis, intrasellar schwannoma, and gangliocytoma can coexist with pituitary adenomas and are referred to as collision sellar lesions [4]. However, concomitant PA and RCC are relatively rare. Our review identified only 40 cases reported to date. In the series of PA complicated with RCC, only a small number of cases have been reported to secrete GH [1, 5–7]. To the best of our knowledge, only 13 cases of patients with GH-producing PA and concomitant RCC have been reported (Table 1) [1, 2, 5–10]. In the present case, PA had been overlooked until noted in the neurosurgical outpatient clinic. Complicated GH-producing PA was initially suspected from the facial appearance of the patient. The difficulty in diagnosing complicated PA was attributable to the location of the RCC. A suprasellar location of the RCC, as in the present case, is rare, with only 3 of 12 previously reported cases of RCC found in the suprasellar region [5, 6, 9]. If RCC is located in the suprasellar lesion, identification of a GH-producing PA is not easy, because an intrasellar lesion may pass unnoticed in such cases. In addition, 3 of 4 cases, including the present case, involved no visual disturbance, and such patients may not receive frequent follow-up of the RCC at the outpatient clinic. In cases where both the GH-producing PA and RCC are located in the sella turcica, it is quite possible that only the RCC will be noticed, since GH-producing PA tends to be much smaller than RCC according to previous reports [4, 7, 8, 11]. At the time of diagnosis of RCC on MRI, it may be useful to perform contrast-enhanced MRI in conjunction with hormonal examination to ensure the rare complication of PA, as in the present case, is not overlooked.

RCC usually appears hyperintense on T1-weighted imaging. However, RCC existing concomitant with PA is known to present with variable intensity on T1-weighted imaging, since it tends to be complicated by hemorrhage in the cyst [1, 13]. In such cases, enhanced MRI is helpful for diagnosis.

When a nonenhanced cyst is demonstrated in cases of PA detected by MRI, the possibility of accompanying RCC should be considered. As for differential diagnosis, RCC usually demonstrates no contrast enhancement of the cyst wall as referred to above and displayed no calcification unlike craniopharyngioma. These radiographic characteristics are helpful in the differential diagnosis of craniopharyngioma.

As for treatments, there is no need to fenestrate RCCs as far as they are asymptomatic. In reported cases of GH-producing PA and concomitant RCC, fenestration of the RCC and surgical biopsy of the cyst wall were performed for 61% [1, 6, 7]. However, GH levels reportedly normalized even in cases without surgical fenestration of the RCC. In the present case, we did not perform surgical biopsy of the cyst wall for fear for CSF leakage, and GH levels decreased to within normal range postoperatively. It is pretty obvious, but it is not necessary to remove cyst wall of RCC to normalize GH. However, if the removal of RCC is needed, endoscopic removal is recommended. Jahangiri et al. said CSF leak did not occur in any of the suprasellar RCCs treated endoscopically, while 14% treated microsurgically experienced a CSF leak. In addition, compared to microsurgery, endoscopy improves rate of complete removal and visual outcomes [11].

In the present case, the appearance of the patient suggested acromegaly and guided us to a diagnosis of RCC with concomitant PA. If the PA in this patient had been nonfunctional, correct diagnosis of the concomitant PA would have been much more difficult. This case offers a reminder that although rare, RCC can accompany PA. We recommend performing contrast-enhanced MRI routinely on suspicion of RCC.

## Figures and Tables

**Table 1:** Summary of Rathke's cleft cyst combined with pituitary adenoma producing growth hormone.

Authors	Age (yrs)	Sex	PA size (mm)	RCC size (mm)	RCC location	RCC T1WI	RCC T2WI	RCC Gd	PA removal	RCC removal	Visual field

Our case	53	M	9	12	Suprasellar	High	Iso.	No	Total	No	Normal

Miyagi et al. [1]	44	M	N/A	N/A	Intrasellar	Low	High	No	Subtotal	Total	Normal

Sumida et al. [7]	67	F	18	22	Intrasellar	Low	High	No	N/A	Removal	Normal
	44	F	28	8	Enclosed	Low	High	No	Removal	No	Normal
	18	M	12	8	Intrasellar	High	High	No	Removal	No	Normal
	46	M	14	7	Intrasellar	Low	High	No	Removal	No	Normal
	56	F	14	13	Intrasellar	High	Low	No	Removal	Removal	Normal
	48	M	15	8	Intrasellar	High	Low	No	Removal	No	Normal

Nishio et al. [6]	44	M	N/A	N/A	Suprasellar	Low	High	No	Subtotal	Removal	Normal
	35	F	N/A	N/A	Suprasellar	Low	High	No	Removal	Removal	Normal
	62	F	21	N/A	Intrasellar	Low	High	No	Removal	Partially	N/A

Lucas et al. [5]	47	F	6	13	Suprasellar	Low	High	No	Removal	Removal	Binasal field defect

Ikeda et al. [12]	50	M	N/A	N/A	Enclosed	Low	High	No	N/A	N/A	Quadrantanopsia

Azarpira et al. [9]	50	F	N/A	N/A	Intrasellar	N/A	N/A	N/A	Removal	Removal	Bilateral temporal hemianopsia

F: female, M: male, N/A: not available, T1WI: T1-weighted image, T2WI: T2-weighted image, Gd: gadolinium, PA: pituitary adenoma, RCC: Rathke's cleft cyst, TR: total removal, unc: unclassified, wk: week, and yr: year.
